# Identification of Subtype-Specific Metastasis-Related Genetic Signatures in Sarcoma

**DOI:** 10.3389/fonc.2020.544956

**Published:** 2020-10-06

**Authors:** Ya-Ling Li, Ya-Li Gao, Xue-Li Niu, Yu-Tong Wu, Yi-Mei Du, Ming-Sui Tang, Jing-Yi Li, Xiu-Hao Guan, Bing Song

**Affiliations:** ^1^Department of Dermatology, The First Hospital of China Medical University, Shenyang, China; ^2^National Health Commission Key Laboratory of Immunodermatology (China Medical University), Shenyang, China; ^3^Key Laboratory of Immunodermatology, Ministry of Education, Shenyang, China; ^4^School of Dentistry, Cardiff University, Cardiff, United Kingdom

**Keywords:** sarcomas, tanespimycin, metastasis, bioinformatics, metastatic

## Abstract

**Background:** Sarcomas are heterogeneous rare malignancies constituting approximately 1% of all solid cancers in adults and including more than 70 histological and molecular subtypes with different pathological and clinical development characteristics.

**Method:** We identified prognostic biomarkers of sarcomas by integrating clinical information and RNA-seq data from TCGA and GEO databases. In addition, results obtained from cell cycle, cell migration, and invasion assays were used to assess the capacity for Tanespimycin to inhibit the proliferation and metastasis of sarcoma.

**Results:** Sarcoma samples (*N* = 536) were divided into four pathological subtypes including DL (dedifferentiated liposarcoma), LMS (leiomyosarcoma), UPS (undifferentiated pleomorphic sarcomas), and MFS (myxofibrosarcoma). RNA-seq expression profile data from the TCGA dataset were used to analyze differentially expressed genes (DEGs) within metastatic and non-metastatic samples of these four sarcoma pathological subtypes with DEGs defined as metastatic-related signatures (MRS). Prognostic analysis of MRS identified a group of genes significantly associated with prognosis in three pathological subtypes: DL, LMS, and UPS. *ISG15, NUP50, PTTG1, SERPINE1*, and *TSR1* were found to be more likely associated with adverse prognosis. We also identified Tanespimycin as a drug exerting inhibitory effects on metastatic LMS subtype and therefore can serve a potential treatment for this type of sarcoma.

**Conclusions:** These results provide new insights into the pathogenesis, diagnosis, treatment, and prognosis of sarcomas and provide new directions for further study of sarcoma.

## Introduction

Sarcomas show a substantial degree of heterogeneity attributable to organ specificity of these cancers. Sarcomas account for ~1% of all adult and 10% of childhood cancers. They can develop in any organ or body site including bone, soft tissue, or skin (1–3). Accordingly, their histopathological characteristics and clinical phenotypes serve as the core for their diagnostic criteria (4). Accurate identification and classification are crucial for successful treatment as responses to specific therapies vary widely among tumor types of different origin and misdiagnosis may lead to poor treatment outcomes (5). However, due to overlap in histopathological features among sarcoma and the existence of multiple subtypes or variations in a single histology, pathologists and clinicians increasingly rely on molecular diagnostic methods (6, 7).

Currently, treatment of sarcoma includes surgical resection, chemotherapy, and radiotherapy; however, the importance of employing a comprehensive treatment regimen involving neoadjuvant chemotherapy and preoperative radiotherapy is increasing (8–10). Despite these advances, life expectancy of patients with metastatic sarcoma remains low. According to the SEER (Surveillance, Epidemiology, and End Results) database of the National Cancer Institute (NCI), sarcoma survival rates vary as a function of the extent of spread, with patients having distant metastases showing a 5-years survival rate of only 16%, while those with partial lesions or partial metastases showing 5-years survival rates of >60%. Due to the extreme degree of heterogeneity within sarcoma, ~100 recognized histopathological subtypes with metastasis-related characteristics have been defined (11).

In this report, we focused on four common sarcoma pathological subtypes including dedifferentiated liposarcoma (DL), leiomyosarcoma (LMS), undifferentiated pleomorphic sarcoma (UPS), and myxofibrosarcoma (MFS). These subtypes were analyzed using TCGA and GEO gene expression data. Differentially expressed genes (DEGs) were identified and grouped in the set of signatures associated with different sarcoma pathological subtypes; metastatic status [metastasis-related signatures (MRS)] and potential responsiveness to treatment were identified. The role of these factors in influencing sarcoma metastasis can provide important information for a more thorough understanding and treatment of this type of cancer.

## Materials and Methods

### Data Acquiring and Preprocessing

Sarcoma-related clinical and RNA-seq data were downloaded using TCGA GDC API. GSE21050 chip data from Affymetrix Human Genome U133 Plus 2.0 Array were downloaded from GEO. TCGA cohort data of sarcoma were preprocessed in the following steps: (1) remove samples without clinical information or PFS (progression-free survival) <30 days, (2) remove data from normal tissue samples, (3) remove genes with 0 FPKM (Fragments Per Kilobase per Million) in greater than half of the samples. The GEO data were preprocessed using the following steps: (1) removal of normal tissue sample data while retaining only data from tumor tissues, (2) converting the metastasis events of PFS data from year or month to days, and (3) using the Bioconductor R package to map the chip probe to the human gene symbol.

### MRS Screening and Functional Annotation

TCGA cohort was grouped according to subtype information to identify the samples as DL, LMS, UPS, and MFS in the metastasis and non-metastasis groups. RNA-seq data were then analyzed using DESeq2. Genes with FDR < 0.05 were defined as MRS. For the GSE21050 dataset, RMA homogenization was performed first and then Limma R package was used for analyses of differences. *P* < 0.001 was required for the DEGs screening threshold. The R package clusterProfiler (v3.8) was used for GO and KEGG functional annotation and enrichment analysis of identified MRS with the *q* value (FDR) of <0.05 as the threshold for significant enrichment.

### Prognostic Analysis

The univariate Cox risk regression model was used to analyze the prognostic relationship between MRS and metastasis events and log rank *p* < 0.05 was set as the significance threshold. The Kaplan–Meier Plotter was used to analyze and display the prognostic curve of sarcoma subtypes transfer events. The R package version 3.5.1 was used for the analyses.

### Drug Discovery

The L1000FWD (http://amp.pharm.mssm.edu/l1000fwd/) platform was used to identify small molecules with potential ability to affect identified DEGs. The L1000FWD database provides record of the up-regulated/down-regulated genes induced by treatment of cancer cell lines with more than 16,000 drugs or small molecules. By comparing the identified up-regulated and down-regulated DEGs to the L1000FWD database record, it can be inferred which small molecular substances may have an effect on identified DEGs. The DGIdb database (http://www.dgidb.org) records more than 40,000 genes and 10,000 drug interactions and provides records of specific genes and drugs associated with interactions of these genes. Based on the DEGs identified, it is possible to identify drugs that have the highest potential to interact with this gene.

Before using CMap (http://portals.broadinstitute.org/cmap/) queries, we used bioconductor R package to convert four subtype MRS gene SYMBOL to Affymetrix probe ID. In the CMap database, drugs with significant negative scores are predicted to become new therapeutic drugs for sarcoma. CMap uses the gene set enrichment analysis algorithm to calculate connectivity scores, with an average score ≤0.65 used to identify potential drug candidates. For each probe generated by CMap ≤-0.67 or >0.67, scores were identified as significant and were used for pathway analysis as an amplitude of ±0.67 represents a twofold change between the treatment and the control.

### Tissue Samples

Samples of sarcoma and adjacent non-tumor tissues (six of the 10 sarcomas were uterine leiomyosarcomas, three liposarcomas, and one undifferentiated pleomorphic sarcoma) were collected from 10 patients (all >16 years of age), immediately placed in liquid nitrogen and preserved at −80°C. None of the sarcoma patients received preoperative anti-tumor therapies. Patients and their families in this study had been fully informed and informed consent was obtained from all participants. This study was approved by the Ethics Committee of Shanghai Tongren Hospital.

### Cell Lines and Culture Conditions

Sarcoma cells, HT-1080 (National Infrastructure of Cell Line Resource 3111C0001CCC000070), and SW-982 (National Infrastructure of Cell Line Resource 3131C0001000700209) were purchased and authenticated originally by the National Infrastructure of Cell Line Resource program using short tandem repeat (STR) analysis; SK-UT-1 cell line was obtained from ATCC (Manassas, VA, USA). Cells were expanded and frozen at low passage within 1 month after receipt from the original stocks. Cells were then thawed and used within 15 passages. Cells were cultured in medium as suggested by the manufacturer supplemented with 10% fetal bovine serum, 100 units/ml penicillin, and 100 μg/ml streptomycin at 37°C in a humidified 5% CO_2_ incubator.

### Cell Proliferation Assay (CCK8 Assay)

For the proliferation assay, sarcoma cells were seeded at 5 × 10^3^ cells/well in 96-well plates and treated with Tanespimycin for 72 h. A 10-μl aliquot of the Cell Counting Kit-8 reagent (Dojindo, Japan) was added to the cells. Following incubation for 3 h, absorbance was measured at 450 nm using a spectrophotometer (Bio-Rad, USA).

### RNA Isolation and qRT-PCR Analysis

RNA extraction from tissues was performed using TRIzol reagent (Invitrogen, Carlsbad, CA, USA). RNA was reverse-transcribed into cDNA with the QuantiTect Reverse Transcription Kit (QIAGEN, Valencia, CA, USA). Real-time PCR analyses were quantified by SYBR-Green (Takara, Otsu, Shiga, Japan), and the levels were normalized to *GAPDH* levels. Sequences of upstream and downstream primers were as follows: *ISG15*, 5′-CGCAGATCACCCAGAAGATCG-3′ and 5′-TTCGTCGCATTTGTCCACCA-3′. *NUP50*: 5′-TCTGGAGGAGGACGCTTTTCT-3′ and 5′-GGGGCACTGGTTATGTTGTTT-3′. *PTTG1*: 5′-ACCCGTGTGGTTGCTAAGG-3′ and 5′-ACGTGGTGTTGAAACTTGAGAT-3′. *SERPINE1*: 5′-ACCGCAACGTGGTTTTCTCA-3′ and 5′-TTGAATCCCATAGCTGCTTGAAT-3′. *TSR1*: 5′-CAGCTCCGAAAGCAGAAGAAG-3′ and 5′-GTTCCAGTGTCCCTATCTTGAAG-3′.

### Immunohistochemistry

Sarcoma samples were fixed in 10% formalin, embedded in paraffin, and processed as 5-μm continuous sections. Samples were dewaxed with ethanol and blocked to inhibit endogenous peroxidase. They were then heated in a microwave to retrieve antigens, cooled to room temperature, and then blocked by incubation in goat serum for 30 min at 37°C. Samples were incubated in rabbit anti-ISG15, anti-NUP50, anti-PTTG1, anti-TSR1, and anti-SERPINE1 (Abcam, Cambridge, UK; 1:1,200) overnight at 4°C, followed by incubation with horseradish peroxidase-coupled goat anti-rabbit secondary antibody at 37°C for 30 min and stained using 3,3′-diaminobenzidine. The cell nucleus was stained blue by hematoxylin. Sections were then dehydrated, cleared by xylene, and mounted. *ISG15, NUP50, PTTG1, TSR1*, and *SERPINE1* expression was detected by IHC using a streptavidin peroxidase method, with adjacent tissues as control groups. The experimental procedure was performed according to the manufacturer's instructions. The Image-ProPlus 6.0 Software (MediaCybernetics, USA) was used to analyze the expression of proteins and statistics on the results of immunohistochemistry.

### Cell Migration and Invasion Assays

Cells were harvested, resuspended in serum-free media, and placed into the upper chamber of a Transwell membrane filter (Corning, NY, USA) for migration assays or in the upper chamber of a Transwell membrane filter coated with Matrigel (Corning) for invasion assays. Culture medium with 10% FBS and 0/5/10 nM Tanespimycin was added to the lower compartment of the chamber to serve as a chemoattractant. After 24 h of incubation, cells were stained with methanol and 0.1% crystal violet, imaged, and counted using an Olympus microscope (Tokyo, Japan).

### Establishment of Single-Cell-Derived Cellular Clones

The cell density was adjusted to 2 × 10^3^/ml, and the cell suspension was 100 μl per well in the 6-well plates per 2 ml of DMEM supplemented with 10% FBS and 100 U/ml penicillin and 100 μg/ml streptomycin. Single-cell-derived clones were grown for 10 days. The culture was pre-cooled three times with PBS, the methanol was fixed for 15 min, the crystal violet dye was applied for 20 min, and the water was rinsed and air dried. The number of visible clones was visually counted and a clone formation rate was calculated: clone formation rate (%) = (clone number/number of inoculated cells) × 100%. This procedure was replicated three times.

### Endothelial Tube Formation Assay

The sarcoma cell culture medium was changed to serum-free DMEM medium for 48 h. Then, cell culture media were collected, centrifuged, and filtered to obtain tumor-conditioned medium (TCM). Matrigel (50 μl; Corning) was added to each well of a 96-well plate and allowed to solidify at 37°C for 30 min. 2 × 10^4^ HUVECs were seeded on the gel with 200 μl of concentrated by ultrafiltration TCM. Then, 5 nM and 10 nM Tanespimycin and equivalent PBS were added and incubated at 37°C. After 8 h, tube formation was observed under a microscope. The tube-like network was visualized by a microscope and photographed. The total tube length and the number of tube branch points were measured using Image-ProPlus 6.0 Software (MediaCybernetics, USA).

### Statistical Analysis

All data were analyzed using the SPSS 21.0 statistical software program (IBM Corporation, Armonk, NY, USA). Graphs were generated with GraphPad Prism 8.0 Software (GraphPad Software, Inc., San Diego, CA). Results are presented as the mean ± standard deviation (SD). Student's *t*-tests, chi-squared tests, or one-way ANOVAs were used where appropriate. A two-tailed *p* < 0.05 was required for results to be considered as statistically significant. *p* < 0.05 was considered as statistically significant (^*^p < 0.05).

## Results

### Sample Information Statistics

**Figure 1:**
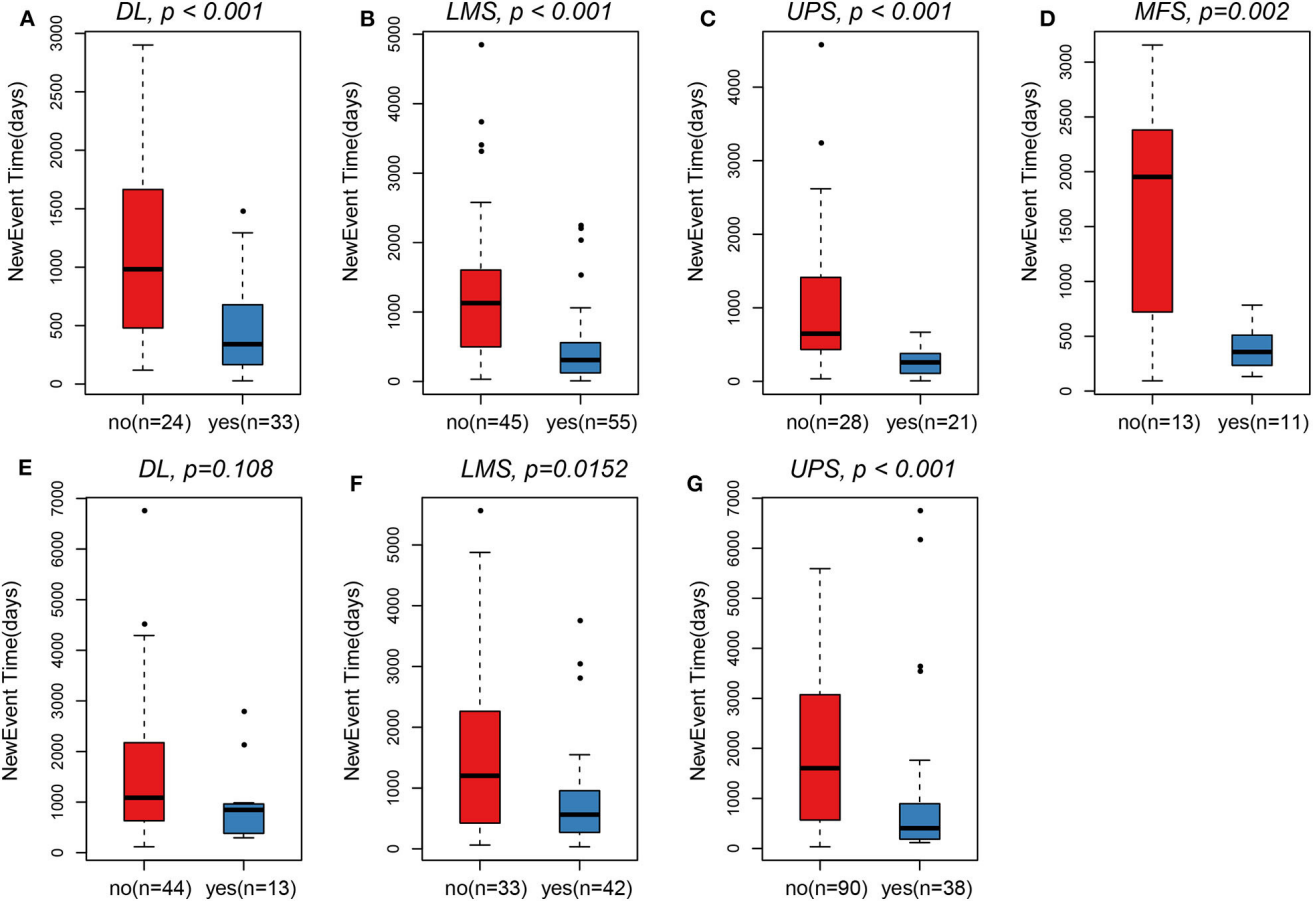
In TCGA, survival time of patients in the metastasis group was observed to be significantly lower than that of patients with non-metastasis. **(A)** DL. **(B)** LMS. **(C)** UPS. **(D)** MFS. In GSE21050, survival time of patients in the metastasis group was also observed to be significantly lower than that of patients with non-metastasis. **(F)** LMS. **(G)** UPS. **(E)** Only pathological subtypes DL did not observe this significant difference.

Clinical sample details are provided in Table 1. A total of 536 samples were obtained, including 250 samples of TCGA and 286 samples of GSE21050. From the sarcoma subtypes identified in the two datasets, the greatest proportions were found to be DL (57/57), UPS (49/128), and LMS (100/75), while other subtypes comprised substantially lower proportions. Overall, the survival of patients with metastases was significantly lower than that of patients with no detectable metastases (*p* < 0.001). In all four pathological subtypes from TCGA cohort, survival time of patients with metastases was observed to be significantly lower than that of patients with no metastases (Figures 1A–D). The same trends were seen for two pathological subtypes LMS and UPS from GSE21050 cohort, but no significant difference was observed for DL subtype (Figures 1E–G).

**Table 1:** Clinical sample details.

	**TCGA**	**GSE21050**
**Metastasis**		
No	120	186
Yes	130	100
Metastasis (PFS)	^*^	^*^
No (mean days)	1229.6	1749.51
Yes (mean days)	453.6	853.41
**Histological type**		
DL	57	57
UPS	49	128
LMS	100	75
MFS	24	
SS	10	
MPNST	8	
DT	2	
Other		26
**Age**		
<50	51	
≥50	199	
**Gender**		
Female	136	
Male	114	

*DL, dedifferentiated liposarcoma; UPS, undifferentiated pleomorphic sarcomas; LMS, leiomyosarcoma; MFS, myxofibrosarcoma; SS, synovial sarcoma; MPNST, malignant peripheral nerve sheath tumors; DT, desmoid tumors*.

* *Indicates means with a significant difference*.

### Identification of MRS

**Figure 2:**
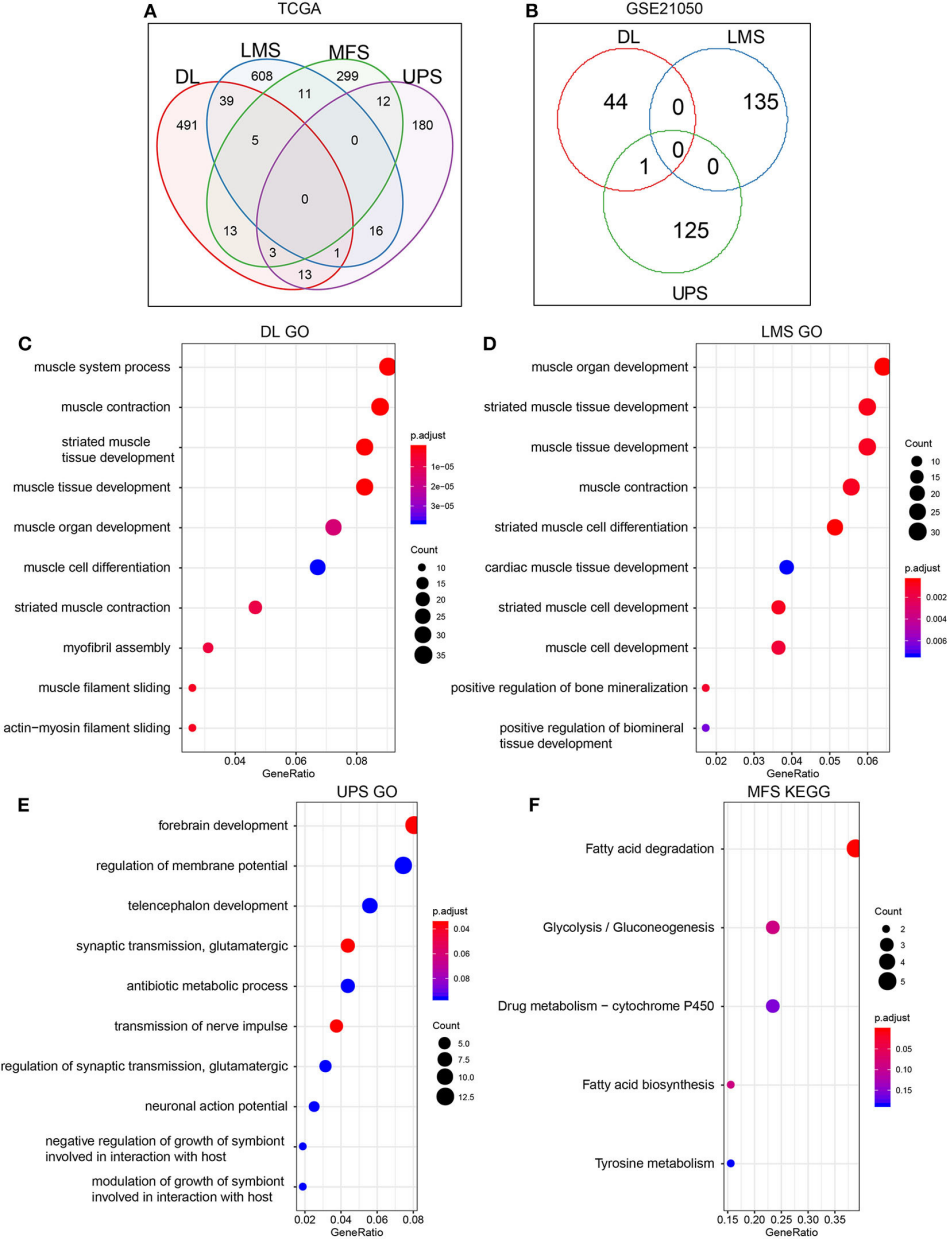
Screening of MRS in metastasis and non-metastasis sarcoma pathological subtypes. **(A)** TCGA. **(B)** GSE21050. Enrichment results of MRS GO Biology Process in THE TCGA dataset of sarcoma pathological subtypes: **(C)** DL. **(D)** LMS. **(E)** UPS. **(F)** The enrichment results of MFS MRS KEGG Pathway (MRS of MFS did not show significant enrichment term on GO, but significant enrichment results were shown on KEGG pathway, so KEGG pathway enrichment results are shown here).

The RNA-seq expression profile data of the TCGA dataset were used to analyze DEGs (Supplementary Tables 1–4) within the four sarcoma pathological subtypes in metastatic and non-metastatic samples. Identified DEGs were defined as MRS. Identified up-regulated DEGs were more abundant than down-regulated DEGs in the metastatic samples. A similar trend was observed in the GSE21050 dataset (Table 2). This indicated that the occurrence of metastasis may be more likely associated with up-regulation of certain pathways rather than inhibition. Comparisons of MRS within different pathological subtypes revealed that there were very few overlapping genes among them (Figures 2A,B). Such findings may be related to the high degree of sarcoma heterogeneity and differences and specificities of metastatic patterns among different pathological subtypes of sarcoma. Enrichment analysis of MRS's GO biology process and KEGG pathway indicated that MRS patterns in DL and LMS were mainly related to muscle tissue homeostasis (Figures 2C,D; Supplementary Tables 5, 6), while MRS patterns in UPS and MFS were related to forebrain development and fatty acid degradation (Figures 2E,F; Supplementary Tables 7, 8).

**Table 2:** MRS statistics.

**Dataset**	**Yes/No**	**DL**	**LMS**	**UPS**	**MFS**
TCGA	Down	89	339	43	224
	Up	476	341	182	119
GSE21050	Down^*^	13	64	38	
	Up^*^	32	71	88	

*Yes indicates transferred and no indicates untransferred*.

* *p < 0.001 was established as the threshold of MRS*.

**Table 3:** The clinical pathological characteristics of sarcoma.

**Age (years)**	
<50	4
≥50	6
**Gender**	
Male	6
Female	4
**TNM stage**	
I–II	2
III–IV	8
**Type of sarcoma**	
Uterine leiomyosarcomas	6
Liposarcomas	3
Undifferentiated pleomorphic sarcoma	1

### Relationship Between MRS and Prognosis

**Figure 3:**
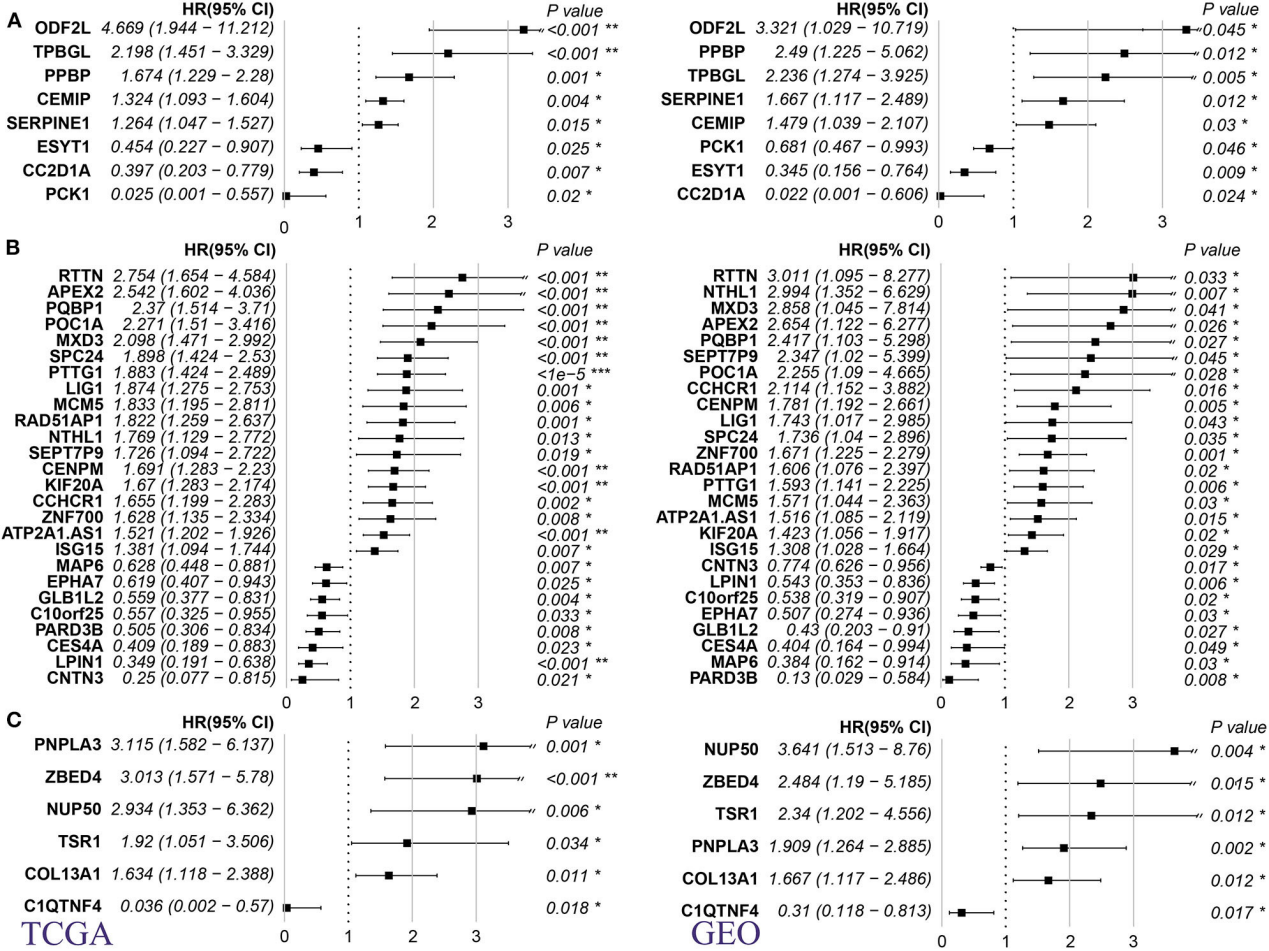
Relationship between MRS of 3 pathological subtypes of sarcoma and prognosis of metastasis. **(A)** DL. **(B)** LMS. **(C)** UPS (Left panel: TCGA dataset; right panel: GSE41258 dataset. HR: hazard ratio, *p-*value is log rank test *p*). *p < 0.05, **p < 0.001, and ***p < 0.0001.

Using TCGA and GSE21050 dataset prognosis information, we analyzed the relationship between MRS and metastatic prognosis of three pathological subtypes, DL, LMS, and UPS, and found 8, 26, and 6 genes (Supplementary Table 9), respectively, that were significantly linked to prognosis. According to HR (hazard ratio) >1 or <1, these genes are divided into adverse prognostic factors and favorable prognostic factors. We found that in the three pathological subtypes described above, these genes are more likely to appear as adverse prognostic factors (HR > 1/HR < 1, Figure 3A, DL: 5/3; Figure 3B, LMS: 18/8; Figure 3C, UPS: 5/1). These findings suggest that the high expression level of these genes has a positive regulatory effect on metastasis and are linked to poor prognosis.

### MRS Genes

**Figure 4:**
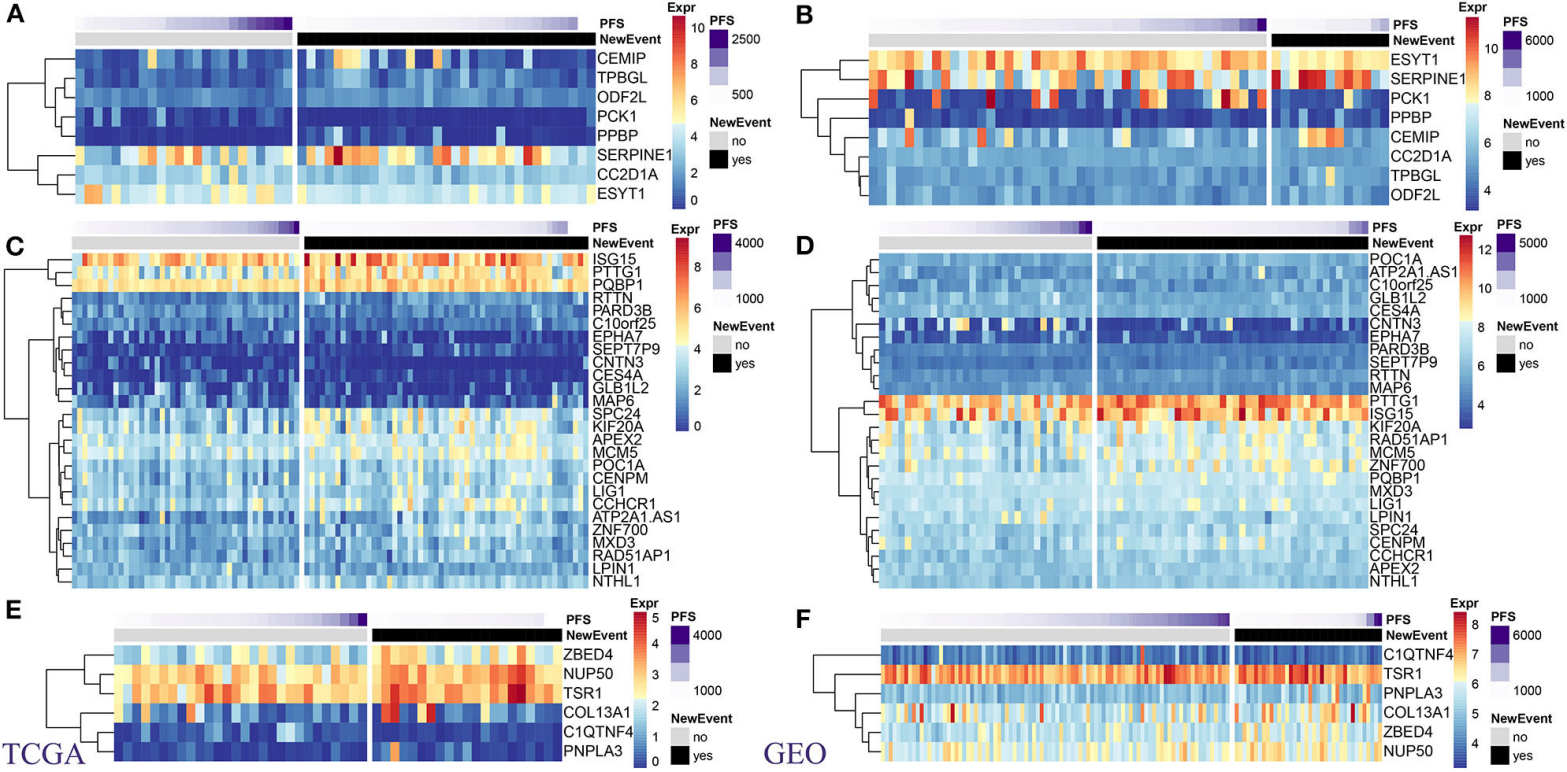
Expression levels of prognostic MRS in pathological subtypes of sarcomas. TCGA: **(A)** DL. **(C)** LMS. **(E)** UPS. GEO: **(B)** DL. **(D)** LMS. **(F)** UPS. (Left panel: TCGA dataset. right panel: GSE41258 data set).

Using TCGA and GSE21050 expression data, we analyzed the expression of MRS as related to prognosis in DL, LMS, and UPS pathological subtypes. Overall, these genes could be aggregated into high- and low-expression groups in both datasets, and a high degree of consistency was present in expression patterns within both datasets (Figure 4). In the DL pathological subtype, the *SERPINE1* gene, which showed the highest expression levels in the TCGA dataset (Figure 4A) and was also highly expressed in the GSE21050 dataset (Figure 4B), and its expression level significantly increased in the metastatic vs. non-metastatic group (Supplementary Table 1). In the LMS pathological subtype, *ISG15* and *PTTG1* showed the highest expression levels in the TCGA (Figure 4C) and GSE21050 (Figure 4D) datasets, and their expression levels in the metastasis group were significantly higher than that in the non-metastasis group (Supplementary Table 2). In the UPS pathological subtype, we found similar expression patterns of *TSR1* and *NUP50* (Figures 4E,F; Supplementary Table 3). These results indicate consistency within identified MRS.

### Potential Drug Analysis

**Figure 5:**
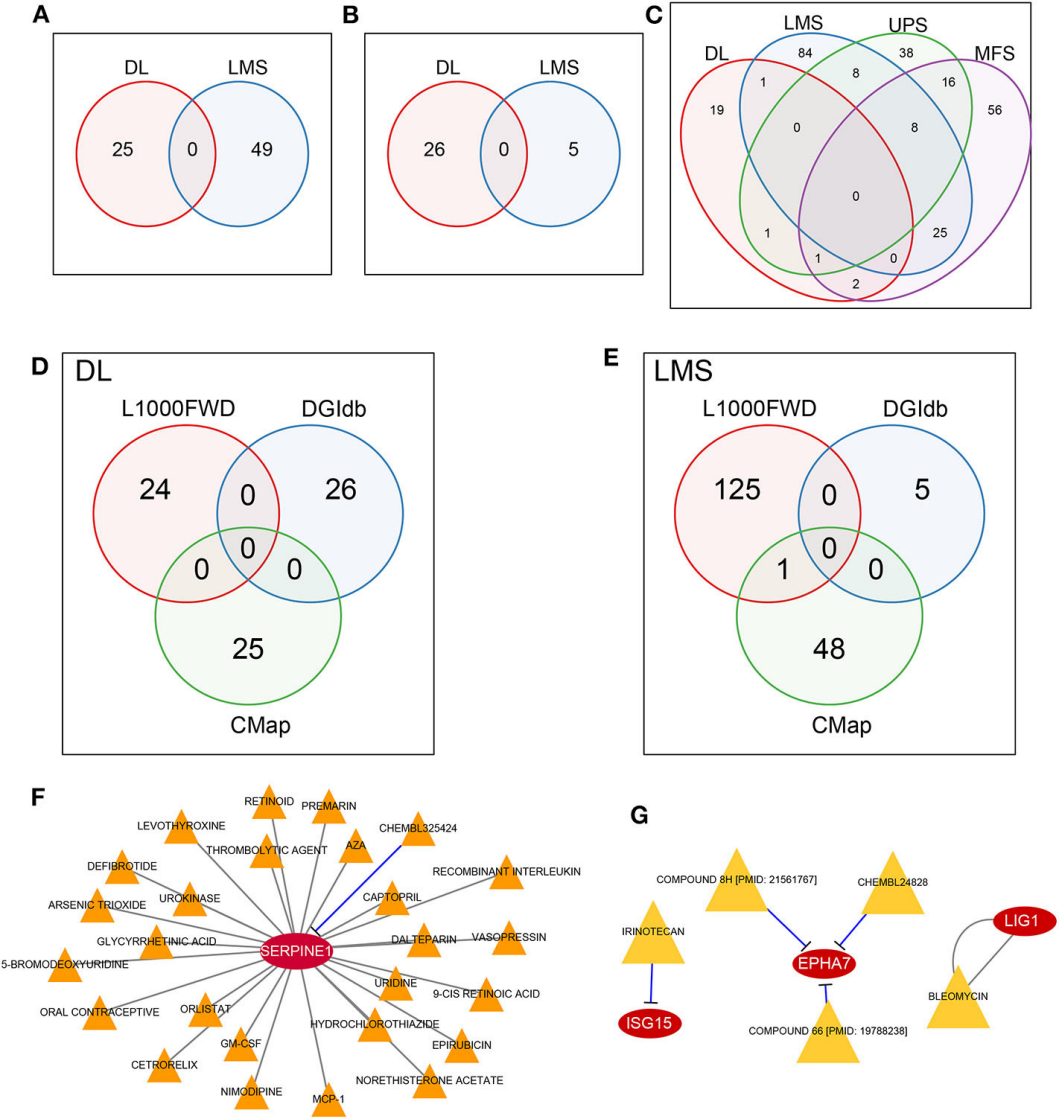
MRS potential drug discovery. **(A)** No overlap between DL and LMS was observed in the L1000FWD database. **(B)** No overlap between DL and LMS was observed in the DGIdb database. **(C)** No overlap of DL, LMS, UPS, and MFS was observed in CMap database. **(D)** DL did not obtain drugs common to L1000FWD, DGIdb, and CMap. **(E)** LMS did not obtain drugs common to L1000FWD, DGIdb, and CMap, but they appeared in L1000FWD and CMap. **(F)** CHEMBL325424 and SERPINE1 in DL pathological subtype are inhibitory relationship. **(G)** In the LMS pathological subtype, IEINOTECAN inhibited ISG15; COMPOUND 8H, CHEMBL24828, and COMPOUND 66 had inhibitory relationships with EPHA7; the relationship between BLEOMYCIN and LIG1 is unknown.

The L1000 database provides records of the effect of more than 16,000 types of drugs and small molecular substances on gene expression patterns within 1,000 tumor cell lines. We used the L1000FWD tool to conduct reverse drug screening for our identified DEGs (including up-regulated and down-regulated genes) in four sarcoma pathological subtypes in the TCGA dataset. We have identified 26 and 49 small molecules with *q*-value <0.05 for DL and LMS subtypes, respectively, while no molecules with significant effect on the UPS and MFS subtypes were identified. Comparing the small-molecule substances with the ability to affect DL and LMS subtypes, no overlapping small-molecule substances were found (Figure 5A, Supplementary Table 10). Similarly, no overlap of small molecules was observed in the DGIdb and CMap databases (Figures 5B,C; Supplementary Tables 11, 12). These results suggest that different sarcoma pathological subtypes will respond differently to drugs, which highlights the importance of drug screening for different pathological subtypes.

For DL and LMS, we compared the potential for drug overlap among L1000, DGIdb, and CMap annotations. For the DL (Figure 5D) subtype, no drugs common for the three databases were found. For the LMS (Figure 5E) subtype, although no drug common to the three databases was found, we did find that the drug Tanespimycin was identified as potentially affecting MRS in LMS in both the L1000 and CMap databases. Tanespimycin is a benzoquinone antitumor antibiotic derived from Geldamycin (12); it can inhibit heat shock protein 90 (HSP90). Inhibition of the HSP90 can promote protein degradation, and this line of therapy is explored for several types of solid tumor or chronic myelogenous leukemias (13–15). Based on the DGIdb database, it was found that in the DL subtype, only the *SERPINE1* gene interacted with a drug, *CHEMBL325424*, that exerted an inhibitory relationship with *SERPINE1* (Figure 5F). Moreover, prognostic analysis revealed that inhibition of *SERPINE1*, which serves as an adverse prognostic factor (Figure 3A, HR > 1) by *CHEMBL325424* may inhibit metastasis in the DL subtype. For the LMS subtype, we obtained drugs that interacted with three genes (Figure 5G); IEINOTECAN inhibited *ISG15*; COMPOUND 8H, CHEMBL24828, and COMPOUND 66 had inhibitory relationships with *EPHA7*; the relationship between BLEOMYCIN and *LIG1* is unknown. *ISG15* and *LIG1* were adverse prognostic factors (Figure 3B, HR > 1), and *EPHA7* was favorable (Figure 3B, HR <1), suggesting that drugs affecting their functions may have a negative effect on the metastasis of LMS subtypes.

### *ISG15, NUP50, PTTG1, SERPINE1*, and *TSR1* Expressions Are Up-Regulated in Sarcoma Tissues

**Figure 6:**
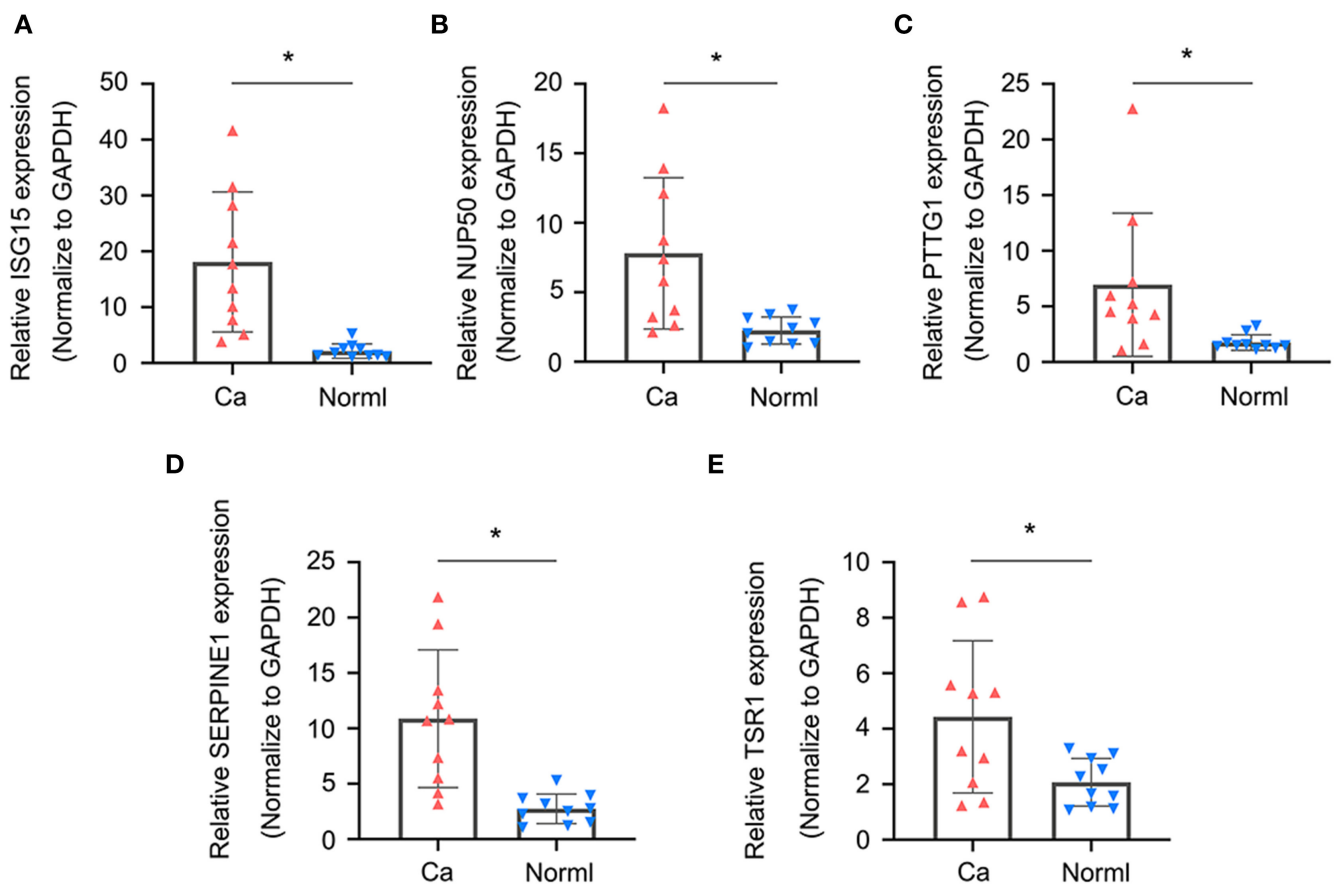
qRT-PCR results indicated that **(A)** ISG15, **(B)** NUP50, **(C)** PTTG1, **(D)** SERPINE1, and **(E)** TSR1 were highly expressed in the sarcoma tissues. (*p < 0.05).

**Figure 7:**
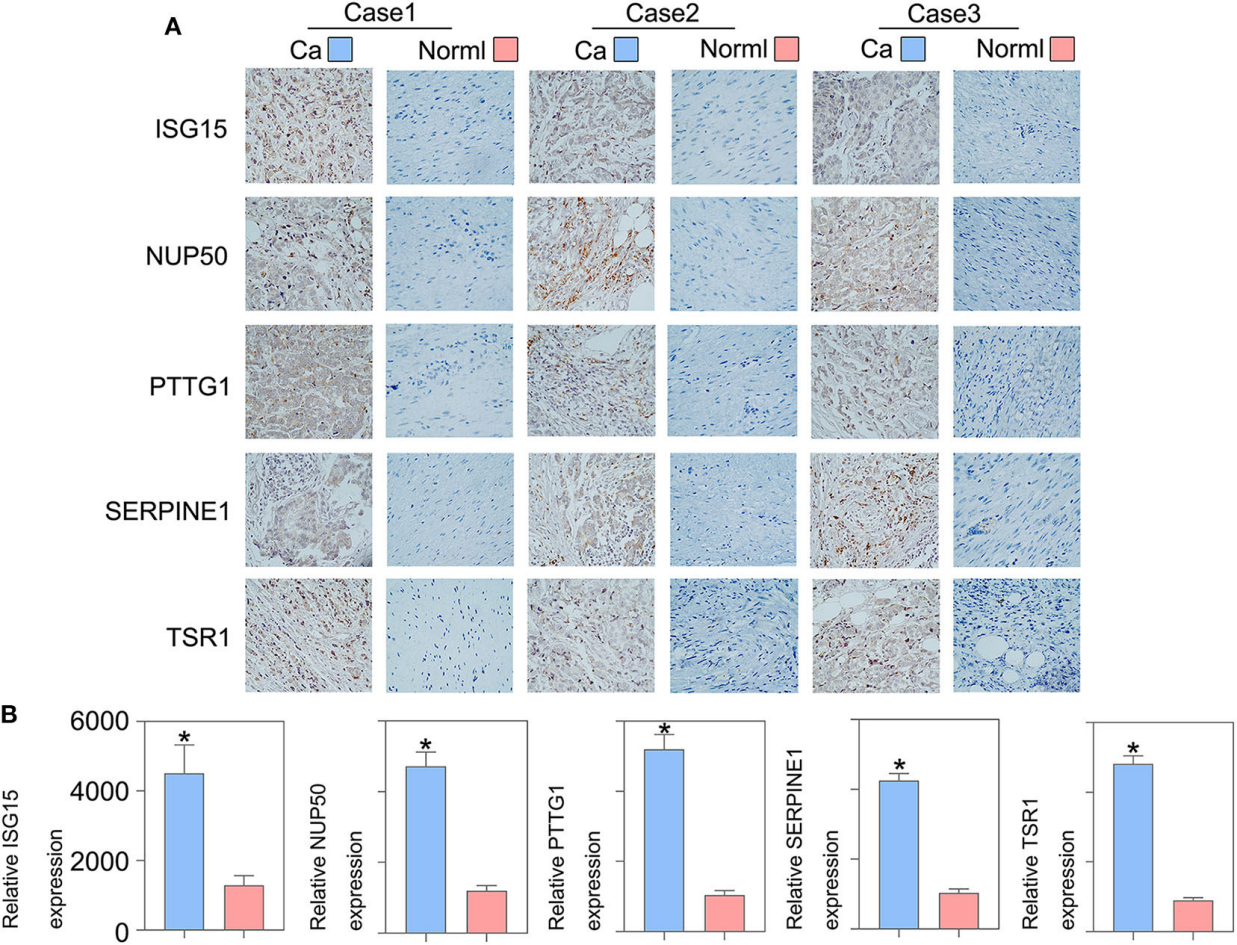
Immunohistochemical results indicated that **(A)** ISG15, NUP50, PTTG1, SERPINE1, and TSR1 were highly expressed in the sarcoma tissues. **(B)** The Image-ProPlus 6.0 Software was used to analyze the expression of proteins (*p < 0.05). Clinical details of patients are shown in Table 3.

To validate whether *ISG15, NUP50, PTTG1, SERPINE1*, and *TSR1* are differentially expressed in sarcoma tissues, we experimentally validated their expression in the tissues of 10 sarcoma patients. The results of qRT-PCR suggested that *ISG15* (Figure 6A), *NUP50* (Figure 6B), *PTTG1* (Figure 6C), *SERPINE1* (Figure 6D), and *TSR1* (Figure 6E) were highly expressed in sarcoma tissues. The results of immunohistochemistry indicated that SG15, NUP50, PTTG1, SERPINE1, and TSR1 were highly expressed in sarcoma tissues (Figure 7A). The Image-ProPlus 6.0 Software (MediaCybernetics, USA) was used to statistically analyze the expression of proteins (Figure 7B) (^*^p < 0.05).

### Tanespimycin Down-Regulates Cell Proliferation, Migration, Invasion, and Angiogenic Ability of Sarcoma Cells

**Figure 8:**
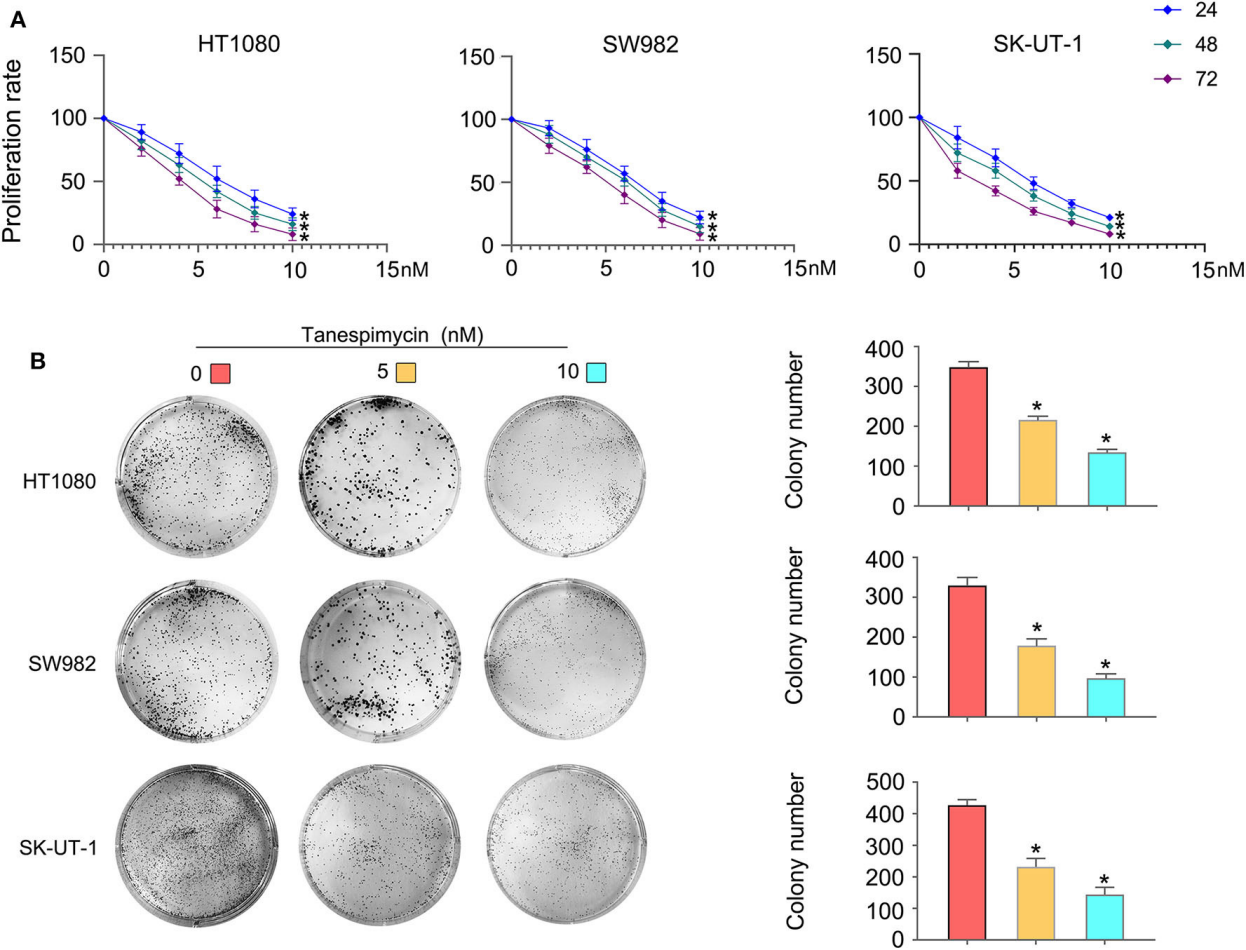
Tanespimycin reduced cell proliferation in a concentration-dependent manner. **(A)** CCK8. **(B)** Colony formation (*p < 0.05).

**Figure 9:**
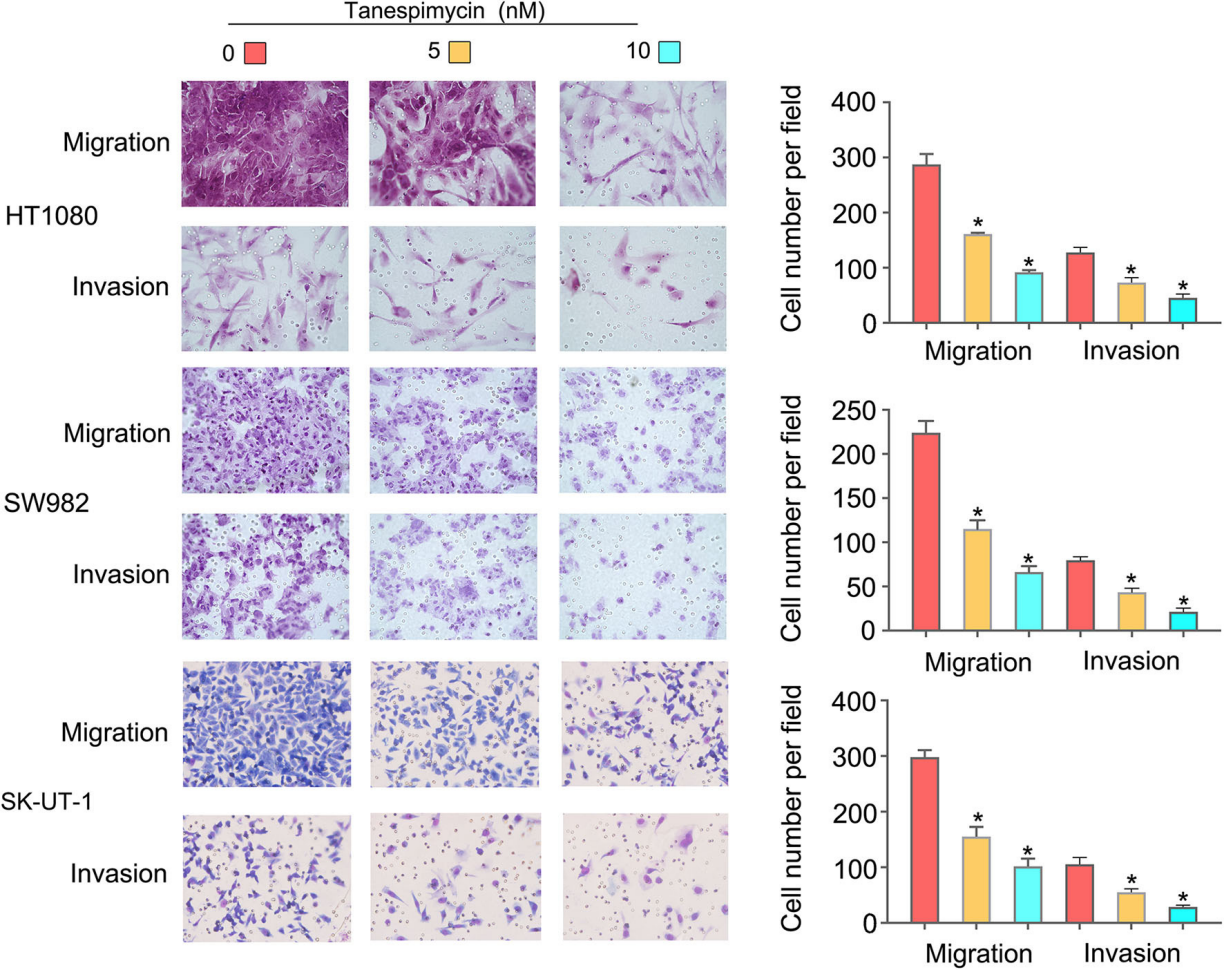
Tanespimycin inhibited the migration and invasion of HT1080, SW982, and SK-UT-1 cells, and the inhibition increased with the increase of concentration (*p < 0.05).

**Figure 10:**
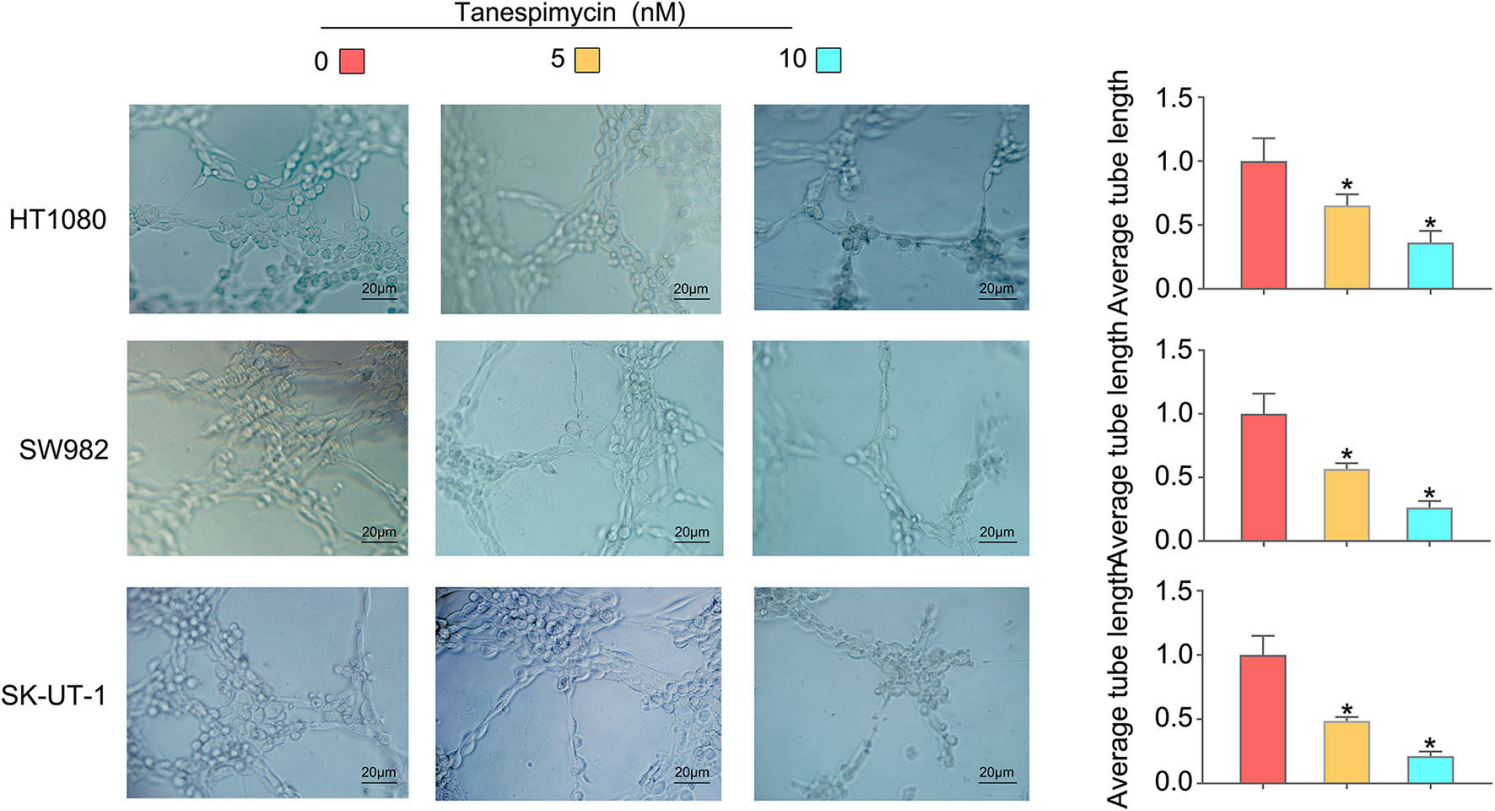
With the increase of Tanespimycin concentration, the proliferation of HUVECs decreased (co-culture with TCM) (*p < 0.05).

Results obtained from both the CCK8 (Figure 8A) and colony formation (Figure 8B) assays revealed that Tanespimycin reduced cell proliferation in sarcoma cell lines in a concentration-dependent manner. Compared with those in the control group, cell migration was obviously inhibited in HT1080, SW982, and SK-UT-1 sarcoma cells after treatment of Tanespimycin in a concentration-dependent manner. Similarly, exposure of Tanespimycin also led to a progressive loss of the invasive ability of HT1080, SW982, and SK-UT-1 cells in a concentration-dependent manner (Figure 9). Tube formation assay was used to assess the effect of Tanespimycin on sarcoma development and the length of tubes was decreased by the higher concentration of Tanespimycin (Figure 10). These data suggest that Tanespimycin is able to reduce the proliferation, migration, invasion, and angiogenic ability of sarcoma cells, and with the higher the concentration, the stronger the effect.

## Discussion

Sarcomas represent a rare tumor type, accounting for ~1% of all tumors most commonly occurring in connective tissue (16, 17). While surgical resection remains the primary treatment for resectable tumors (18), the combination of preoperative (preRT) or postoperative (PORT) radiotherapy and chemotherapy can also play a role (19). In this study, we analyzed gene expression data of 536 sarcoma samples from TCGA and GEO (GSE21050) databases. The survival time of the patients diagnosed with sarcomas classified as one of the four pathological subtypes DL, LMS, UPS, and MFS in the metastasis group and the non-metastasis group was analyzed. The survival of the patients with metastases was significantly lower than for patients with no detectable metastases. When comparing the DEGs in metastatic vs. non-metastatic samples of the four pathological subtypes, a set of genes related to metastasis development was identified as MRS. Few MRS were found to be common among the different pathological subtypes, indicating that differences in metastasis patterns were present within the different pathological subtypes. Results obtained from functional annotations of MRS revealed that MRS in DL and LMS were mainly related to biological processes of muscle tissue, while MRS in UPS and MFS were related to forebrain development and fatty acid degradation. Prognostic analysis of MRS further identified a group of genes that were significantly associated with prognosis. These results indicated that the genes in the three pathological subtypes DL, LMS, and UPS were more likely to be linked with adverse prognostic factors, suggesting that the high expression levels of identified genes exerted a positive regulatory effect on metastasis, thus accounting for their association with poor prognosis. Public drug databases such as L1000, DGIdb, and CMap, were then used to identify a set of potential drugs (small-molecule substances) with potential to affect identified MRS. Tanespimycin was identified in both L1000 and CMap databases, suggesting that it might serve as a drug to treat metastasis of the LMS subtype. To verify these findings, qRT-PCR, immunohistochemistry, transwell, cell proliferation, and neovascularization assays were performed. Taken together, results of these experiments suggest that high expression levels of *ISG15, NUP50, PTTG1, SERPINE1*, and *TSR1* exert a positive regulatory effect on metastasis, suggesting poor prognosis, and that Tanespimycin may effectively treat LMS sarcoma subtype.

The *ISG15* transcription is abnormally expressed in most human malignancies and exerts both pro- or anti-tumor functions (20). *ISG15* is considered as a tumor biomarker due to its high level of expression as confined to tumors (21), and sensitivity to the anti-cancer drug camptothecin (Topotecan) (22). *ISG15*, via the c-MET/Fyn/beta-catenin pathway in esophageal squamous cell carcinoma, shows tumor-promoting effects (23). Another gene identified in this study was *NUP50*. *NUP50* can form a protein cluster with *XPO1*, which is inhibited by bortezomib, showing inhibiting activity in multiple myeloma (24). *PTTG1* has also been associated with the occurrence of tumors. Increased expression of the nuclear protein in *PTTG1* was observed in malignant adrenal tumors, and *PTTG1* can serve as a biomarker for this tumor type (25). Overexpression of *PTTG1* is an independent prognostic factor for colorectal cancer patients, and knockout of *PTTG1* can inhibit the growth and metastasis of colorectal cancer (26). Moreover, *PTTG1* promotes the invasion of esophageal squamous cell cancer cells by inducing epithelial–mesenchymal transformation, and *PTTG1* is involved in inducing epithelial–mesenchymal transformation by activating the expression of *GLI1* in esophageal squamous cell carcinoma (27). Results from a number of studies have shown that *SERPINE1* can be used as a prognostic biomarker for gastric cancer (28–30). Down-regulation of *SERPINE1* can inhibit the growth of glioma *in vivo*, and up-regulation of mir-1275 activates the p53 signaling pathway by regulating *SERPINE1*, thus inhibiting the proliferation, invasion, and migration of glioma cells (31). Finally, *ADAMTS5*'s recombinant *TSR1* inhibits the growth of melanoma in mice by anti-angiogenesis mechanism (32), and *ADAMTS5* can also be used as a prognostic biomarker for prostate cancer (33).

To our knowledge, there is no literature suggesting that Tanespimycin can be used to treat sarcomas. It has been reported that Tanespimycin is a classical inhibitor of Hsp90 (34), and Runx2 is transcriptionally regulated by Hsp90 through the AKT/gsk-3/catenin signaling pathway, leading to apoptosis of osteosarcoma cells (35). Our current results suggest that Tanespimycin inhibits the proliferation and metastasis of sarcomas *in vitro*.

In summary, here we demonstrate that *ISG15, NUP50, PTTG1, SERPINE1*, and *TSR1* are highly expressed in sarcoma tissues and associated with sarcoma metastasis and poor prognosis. In addition, we present the first evidence indicating that Tanespimycin inhibits the proliferation and metastasis of sarcomas. Taken together, these results provide new insights into the pathogenesis, diagnosis, treatment, and prognosis of sarcomas and provide new directions for further study of sarcomas.

## Data Availability Statement

The original contributions presented in the study are included in the article/Supplementary Material, further inquiries can be directed to the corresponding author/s.

## Ethics Statement

This study was approved by the Ethics Committee of Shanghai Tongren Hospital. Written informed consent to participate in this study was provided by the patient and patient's legal guardian/next of kin.

## Author Contributions

Y-LL made substantial contributions to the conception and design, acquisition, analysis, and interpretation of data. BS and X-HG were involved in drafting the manuscript and revising it critically for important intellectual content. Y-LL, Y-LG, X-LN, Y-TW, Y-MD, M-ST, J-YL, X-HG, and BS gave final approval of the version to be published. All authors contributed to the article and approved the submitted version.

## Conflict of Interest

The authors declare that the research was conducted in the absence of any commercial or financial relationships that could be construed as a potential conflict of interest.
